# Effects of presentation level on speech-on-speech masking by voice-gender difference and spatial separation between talkers

**DOI:** 10.3389/fnins.2023.1282764

**Published:** 2023-12-14

**Authors:** Yonghee Oh, Phillip Friggle, Josephine Kinder, Grace Tilbrook, Sarah E. Bridges

**Affiliations:** ^1^Department of Otolaryngology-Head and Neck Surgery and Communicative Disorders, University of Louisville, Louisville, KY, United States; ^2^Department of Speech, Language, and Hearing Sciences, University of Florida, Gainesville, FL, United States

**Keywords:** speech segregation, voice gender release from masking, spatial release from masking, level, presentation level, speech-on-speech masking

## Abstract

Many previous studies have reported that speech segregation performance in multi-talker environments can be enhanced by two major acoustic cues: (1) voice-characteristic differences between talkers; (2) spatial separation between talkers. Here, the improvement they can provide for speech segregation is referred to as “release from masking.” The goal of this study was to investigate how masking release performance with two cues is affected by various target presentation levels. Sixteen normal-hearing listeners participated in the speech recognition in noise experiment. Speech-on-speech masking performance was measured as the threshold target-to-masker ratio needed to understand a target talker in the presence of either same- or different-gender masker talkers to manipulate the voice-gender difference cue. These target-masker gender combinations were tested with five spatial configurations (maskers co-located or 15°, 30°, 45°, and 60° symmetrically spatially separated from the target) to manipulate the spatial separation cue. In addition, those conditions were repeated at three target presentation levels (30, 40, and 50 dB sensation levels). Results revealed that the amount of masking release by either voice-gender difference or spatial separation cues was significantly affected by the target level, especially at the small target-masker spatial separation (±15°). Further, the results showed that the intersection points between two masking release types (equal perceptual weighting) could be varied by the target levels. These findings suggest that the perceptual weighting of masking release from two cues is non-linearly related to the target levels. The target presentation level could be one major factor associated with masking release performance in normal-hearing listeners.

## Introduction

Listeners often have difficulty focusing on the auditory signal from the speaker of interest, or “the target,” when they are surrounded by multiple voices conveying informational speech in multi-talker listening environments. The “cocktail party” phenomenon (Cherry, 1953), also referred to as “speech-on-speech masking,” is a result of these communication challenges in multi-talker listening situations. All listeners in multi-talker listening situations are impacted by “speech-on-speech masking,” which is made worse by deficiencies in auditory perception and processing brought on by hearing loss and/or aging (Helfer and Freyman, 2008). In order to enhance the perception of those with auditory deficits, we must first understand the strategies utilized in these situations by normal hearing (NH) listeners. In many previous studies with NH listeners, the talkers in multi-talker situations (i.e., speech-on-speech masking situations) can be segregated by utilizing two major acoustic cues: (1) differences in voice characteristics (e.g., pitch, timbre, and loudness) among talkers (Brungart, 2001; Brungart et al., 2001; Darwin et al., 2003; Mackersie et al., 2011; Byrne et al., 2022) and (2) spatial separation between target and competing talkers (Ericson et al., 2004; Best et al., 2012; Gallun et al., 2013; Srinivasan et al., 2016; Humes et al., 2017). It should be noted that the aforementioned cues allow the listener a “release from masking” which refers to the ability to separate non-target speech from mixed voices and understand the target speaker.

Masking release is greater when target and masker talkers are different genders than when they are the same gender, referred to as “Voice Gender Release from Masking” (VGRM). This may be related to differences in voice characteristics such as fundamental frequency (F0) and vocal-tract length (VTL), which correlate with the speaker’s birth gender/sex (Darwin et al., 2003). It should be noted that the term “gender” denotes the classical categorization of a talker’s voice with their assigned sex at birth. In this study, we will refer to these benefits from talker sex differences as voice gender release from masking (VGRM), as referred to in the previous studies (Oh et al., 2021, 2022). Brungart and his colleagues reported the importance of voice characteristics in speech segregation in multi-talker listening situations (Brungart et al., 2001). Their studies showed that the percentage of correctly identified targets increased by 15% to 20% points when the target and maskers were of different genders compared to situations when they were of the same gender (Brungart, 2001; Brungart et al., 2001). Other studies investigated the relative influence of F0 and VTL on speech-on-speech masking performance (Darwin et al., 2003; Mackersie et al., 2011). According to those investigations, talker differences produced by parametric manipulation of F0 or VTL alone resulted in masking release; however, performance increases associated with these singular manipulations were less than the performance increases observed with the full complement of acoustic cues of natural voices. These findings indicate that both F0 and VTL cues can contribute interdependently to masking release performance.

“Spatial Release from Masking” (SRM) is facilitated by increasing differences in the apparent source location of the target and competing speech. Most previous SRM studies showed that listeners can achieve significant SRM (up to 18 dB) in a variety of different spatial configurations, but the amount of SRM can be affected by various factors: (1) subject factors such as age and hearing loss (Best et al., 2012; Srinivasan et al., 2016; Humes et al., 2017; Jakien et al., 2017); (2) stimulus factors such as stimulus presentation level, the number of competing talkers, and target and masker similarity (Brungart et al., 2001; Kidd et al., 2010; Eddins and Liu, 2012; Humes et al., 2017; Jakien et al., 2017). In general, less spectral or temporal overlap between target and masker speech can yield greater SRM (i.e., energetic masking). Confusion of masker speech content with target speech content can reduce SRM (i.e., informational masking).

The interaction of voice characteristic differences and spatial separation between talkers has been explored in previous studies (Ericson et al., 2004; Allen et al., 2008; Oh et al., 2021, 2022). First, Ericson et al. (2004) reported that the speech-on-speech masking performance improved by 25% to 35% when same-sex competing talkers were separated spatially by ±45°, and the improvement is roughly equivalent to replacing the same-sex interfering talkers with different-sex interfering talkers (~102-Hz F0 differences). This result implies that the masking release by VGRM and SRM could be equally weighted at ±45° separation between target and maskers. Second, Allen et al. (2008) reported that the VGRM and SRM were roughly equal at 10 to 13 dB at the ~79-Hz F0 difference and ± 30° spatial separation between talkers, and the spatial separation to make the equal perceptual weighting between VGRM and SRM could show inter-subject variability. More recently, Oh and his colleagues systematically explored this perceptual weighting related to the subject’s utility of two cues, and their results demonstrated that the masking release by VGRM and SRM elicits an unequal perceptual weighting, and the magnitude of masking release is the same for the two cue types (e.g., equal perceptual weighting) at the ~104-Hz F0 difference and the ~±15° spatial separation between target and masker (Oh et al., 2021). Those results indicate that talkers’ small spatial differences cause a greater perceptual weighting of VGRM compared to SRM. Likewise, larger spatial separation increases the perceptual weighting of SRM in comparison to VGRM.

It should be noted that all references mentioned above used the coordinate response measure (CRM; Bolia et al., 2000) speech corpus, which is one of the English speech corpora for measuring masking release performance. In addition, most of the references above have focused primarily on the benefits of voice-gender difference and/or spatial separation cues in fixed stimulus presentation level conditions, but there is also evidence that the target presentation level could change the effectiveness of speech-on-speech masking. According to Guthrie and Mackersie (2009), target-presentation levels could affect a listener’s maximal suprathreshold speech recognition ability (i.e., by the selection of sensational levels across the range of hearing losses). In the study by Arbogast et al. (2005), for the specific example of the masking release by talker’s spatial separation (SRM), NH individuals could have about a 6-dB benefit on their SRM performance (between 0° and 90° target-masker separation) when the masker sensational level (SL) increased from 25.8 to 38.8 dB SL. Similarly, the study by Jakien et al. (2017) showed that NH listener’s SRM improved (2–3 dB between 0° and 45° target-masker separation) by increasing target-presentation levels (20 to 40 dB SL). In addition, Brungart (2001) found that speech-on-speech masking performance is non-linearly changed by the relative levels of target and masker speech when talkers’ voice characteristic differences are taken into account. In other words, the listener’s speech-on-speech masking performance (measured by psychometric function) is not always monotonic (“S”-shaped); instead, the psychometric function could flatten or even become “N”-shaped. This implies that target speech near masker levels could result in a higher percentage of correct identifications than target speech at either a lower or higher level relative to masker speech, or vice versa. The researchers hypothesized that this level-dependent change in masking release may be due to a limit on audibility to access both monaural and binaural cues required for separating competing talkers or due to the different roles of informational and energetic masking in the perception of competing speech messages.

Therefore, how the target-presentation level specifically influences speech-on-speech masking performance in a level-dependent manner remains to be carefully determined. The goal of this study was to explore the effects of presentation level on a NH listener’s speech recognition abilities in multi-talker listening situations. To the best of our knowledge, the effect of stimulus presentation level on masking release has not been investigated when both voice and spatial cues are accounted for together. We hypothesize that target level may non-linearly alter the listener’s use of either the talker’s voice-gender differences cue or the spatial separation cue in multi-talker listening situations. If the non-linear level-dependent changes were observed, then, we could further identify interactions between voice and spatial cues, yielding an understanding of the effects of target levels on the perceptual weighting between the two major cues in speech-on-speech masking performance.

## Materials and methods

### Subjects

Sixteen paid young adult NH listeners (10 females, mean age = 23.7 ± 2.1 years old) participated in this study. NH was defined as audiometric thresholds <25 dB hearing level (HL) at octave frequencies between 125 and 8,000 Hz. Average thresholds across the frequencies were 3.8 (± 7.1) dB HL for the left ear and 4.2 (± 6.4) dB HL for the right ear. All subjects scored ≥27 on the Mini-Mental State Examination (MMSE; Folstein et al., 1975), ruling out cognitive impairment that would potentially influence performance. All experiments were conducted according to the guidelines for the protection of human subjects as set forth by the Institutional Review Board (IRB) of the University of Florida, and the methods employed were approved by that IRB.

### Stimulus materials

Speech sentences were drawn from the Coordinate Response Measure (CRM; Bolia et al., 2000) speech corpus, one of the most popular English speech corpora for measuring SRM. Each sentence of the CRM speech corpus consists of the same syntactic structure in the form: “Ready [*call sign*] go to [*color*] [*number*] now.” There are eight *call signs* (“Arrow,” “Baron,” “Charlie,” “Eagle,” “Hopper,” “Laker,” “Ringo,” “Tiger”), four *colors* (“blue,” “green,” “red,” “white”), and eight *numbers* (1–8). The corpus includes all possible *call sign*, *color*, and *number* combinations spoken by four male (F_0_ = 100 ± 7 Hz) and four female talkers (F_0_ = 204 ± 12 Hz), leading to 2,048 unique speech samples (256 CRM phrases for each talker). Note that fundamental frequency (F0), which represents the voice pitch, was estimated using the cepstrum algorithm in MATLAB where the output is the Fourier transform of the log of the magnitude spectrum of the input waveform (Flanagan, 1965). F0 for each talker was averaged across all of that talker’s CRM speech stimuli.

### Procedure

**Figure 1:**
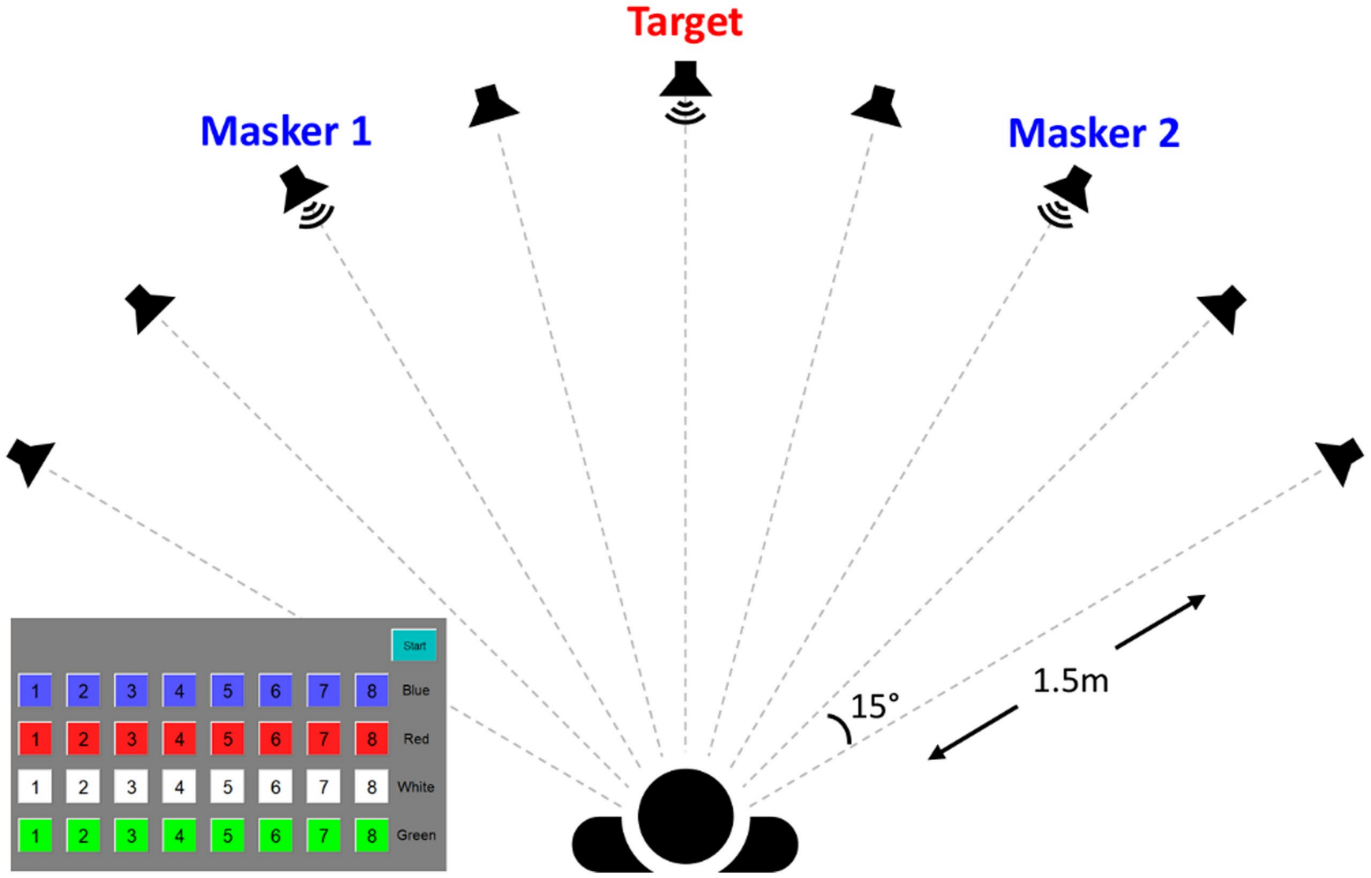
Schematics of the experimental setup, which illustrates an example ±30° target-masker spatial separation and a screenshot of the touchscreen choices.

The experiment was conducted in a single-walled, sound-attenuating booth. Speech stimuli, stored as .wav files with a sampling rate of 44.1 kHz, were presented using custom MATLAB (version R2018b, MathWorks, Massachusetts, United States) scripts. Stimuli were routed through an RME UFX+ audio interface (RME Audio, Haimhausen, Germany) and delivered via frequency-equalized Yamaha HS5 loudspeakers (Yamaha, Shizuoka, Japan). A total of 9 loudspeakers were separated by 15° in the horizontal plane and positioned in the front hemifield a distance of 1.5 m from the center of the listener’s head (See the speaker array configuration illustrated in Figure 1). The output of all loudspeakers was calibrated using a Brüel and Kjær sound level meter with an A-weighting filter (Brüel and Kjær Sound and Vibration Measurement A/S, Nærum, Denmark).

Prior to the main speech-on-speech masking experiment, the baseline speech reception threshold (SRT) was measured in quiet (target only, no masker). The listeners were presented with a target phrase from the CRM corpus with the call sign “Charlie” played from the speaker at 0° azimuth. Here, the target phrase was randomized from four female talkers at each trial. After each stimulus presentation, listeners selected the key words (*color* and *number*) in the target phrase from a grid of 32 possible color/number combinations displayed on an iPad tablet computer (Apple, Cupertino, California, United States). Please see the subject response screen illustrated in Figure 1. Immediate feedback was provided at the top of the grid-array with text reading “correct” or “incorrect.” During the experiments, listeners were instructed to face the front speaker and attend to the target sentence during all experiments. A one-up, one-down, adaptive procedure (Levitt, 1971) was used to estimate the 50% correct point on the psychometric function. The initial target level was set at 65 dBA and decreased in level by 5 dB for each correct response until an incorrect response occurred, then increased in level for each incorrect response until a correct response, and so on. This was repeated until the adaptive track had three reversals, at which point the step size was reduced to 1 dB and six more reversals were obtained. The threshold calculation was based on the average of the last six reversals. The threshold was computed as the average across the two separate runs. The averaged in-quiet SRT was 18 ± 2.8 dBA.

Following threshold measurements in quiet, speech recognition thresholds in the competing speech were measured at three different target levels (30, 40, and 50 dB SL), defined relative to each listener’s in-quiet SRT. Each listener was presented with three simultaneous phrases from the CRM corpus (1 target phrase and 2 simultaneous masker phrases). The task was to report the key words (*color* and *number*) in the target phrase, which was indicated by the call sign “Charlie.” Similar to the quiet condition, the target phrase was randomized from four female talkers and presented at a fixed 0° azimuth. The masker phrases had exactly the same form as the target but a different *call sign*, *color*, and *number*, randomly selected from the same- or different-gender talkers on each trial according to the target-masker gender combinations. To prevent confusion, no maskers contained the call sign “Charlie” and none of the phrases had common color or number key words on a single trial. For the spatial configuration between target and maskers, the presentation of the two masker phrases was either colocated with the target phrase (0°) or from left and right loudspeakers at progressively greater spatial separations (±15°, ±30°, ±45°, and ± 60°). Symmetric target-masker separation minimized the availability of any better ear cue due to the head shadow effect (Shaw, 1974; Marrone et al., 2008) and thus maximized the potential to use spatial cues or voice cues for source segregation. In addition to the five spatial separations, two different voice gender target-masker combinations were tested: FF (female target/female maskers: same target-masker gender condition) and FM (female target/male maskers: different target-masker gender condition). To improve reliability, each condition was tested twice, for a total of 3 target levels × 5 spatial separations × 2 gender combinations × 2 repetitions = 60 separate measures. All gender differences and spatial configurations were tested in a random order to reduce any listener predictability.

The target from the CRM corpus was fixed at three different levels (30, 40, and 50 dB SL). The presentation level of the combined masker sound was adjusted after each trial using a one-up, one-down, adaptive procedure (Levitt, 1971) to estimate the masker level yielding 50% correct recognition of both target *color* and *number*. The initial level for the masker sentence was set at 30 dB below the target level and increased in level by 5 dB for each correct response until an incorrect response occurred, then decreased in level for each incorrect response until a correct response, and so on. This was repeated until the adaptive track had three reversals in direction, at which point the step size was reduced to 1 dB and six more reversals were obtained. The threshold masker level for a given block of trials was estimated as the average of the last six reversals, and the target-to-masker ratio (TMR) was calculated by subtracting the masker level from the target levels. The threshold for each condition was computed as the average across the two separate runs. It should be noted that all listeners responded correctly to the first trial of the adaptive tracks at all three target levels (30, 40, and 50 dB SL), indicating that the three target levels tested in this study were audible enough for listeners to identify the target keywords in the quiet condition. All statistical analyses were conducted in SPSS (version 25, IBM).

## Results

**Figure 2:**
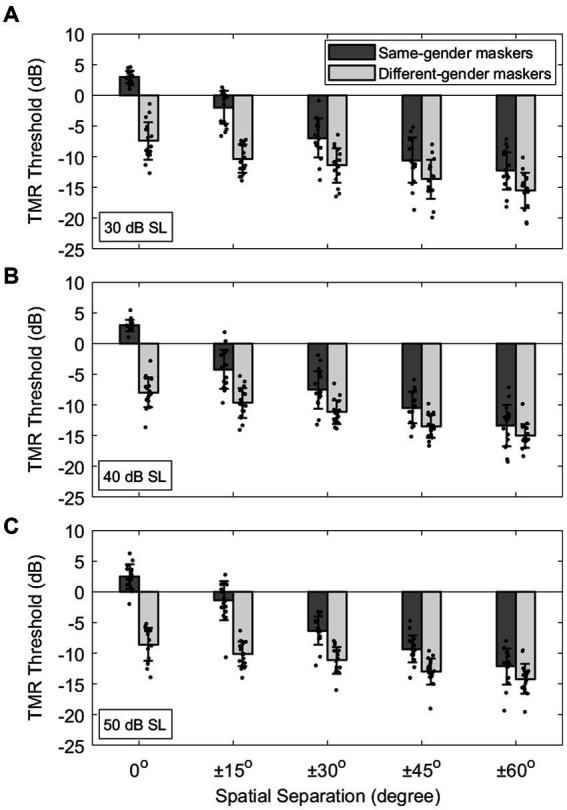
**(A-C)** Individual and average target-to-masker ratio (TMR) thresholds as a function of target-masker spatial separation (0°, ±15°, ±30°, ±45°, ±60°) at three different target levels (30, 40, and 50 dB SL). Error bars represent standard deviation of the mean.

Figure 2 shows individual and mean TMR thresholds (±1 standard deviation around the mean) on the ordinate as a function of the target-maskers spatial separation on the abscissa for the three target-presentation levels: 30, 40, and 50 dB. Note that smaller, or more negative, TMR thresholds indicate better (or improved) speech recognition ability. The results show that, for all target levels, the different target-masker gender conditions (light gray bars) elicited lower TMR thresholds compared to the same-gender conditions (dark gray bars). Those TMR threshold improvements are indicative of VGRM and are maximized at the co-located (0°) spatial condition. The results also show that spatial separation of the maskers from ±15° to ±60° (left to right) relative to the target at 0° led to smaller TMR thresholds. Those improvements are indicative of SRM and are maximized between the 0° and ±60° spatial conditions. A linear mixed model (LMM) analysis was used to analyze the data with the TMR threshold as a dependent variable, the target level (30, 40, and 50 dB), spatial separation (0°, ±15°, ±30°, ±45°, and ± 60°) and voice-gender difference (same-gender and different-gender) between target and makers as fixed factors, and the subject as a random factor. The LMM results showed significant effects of all three fixed factors (target level: *F*_2,435_ = 4.08, *p* = 0.018; spatial separation: *F*_4,435_ = 507.76, *p* < 0.001; voice-gender difference: *F*_1,435_ = 1029.05, *p* < 0.001) as well as significant interactions between target level and voice-gender difference (*F*_2,435_ = 4.05, *p* = 0.018) and between spatial separation and voice-gender difference (*F*_4,435_ = 79.58, *p* < 0.001).

The primary goal of this study was to explore the effects of presentation level on masking releases due to (1) the voice-gender differences between talkers (i.e., VGRM), (2) the spatial separation between talkers (i.e., SRM), and (3) both VGRM and SRM. The amounts of VGRM and SRM were computed from the TMR thresholds at each target level condition as follows: VGRMs account for gender-difference benefits (i.e., FF_k_ − FM_k_), SRMs account for spatial separation benefits (FF_0_ − FF_k_), and combined release from masking (VGRM + SRM) accounts for benefits from gender difference and spatial separation (FF_0_ − FM_k_). Here, the subscript “k” indicates target and masker spatial separation (k = 0°, ±15°, ±30°, ±45°, or ± 60°), and the “FF” and “FM” indicate same-gender and different-gender target-masker conditions, respectively.

**Figure 3:**
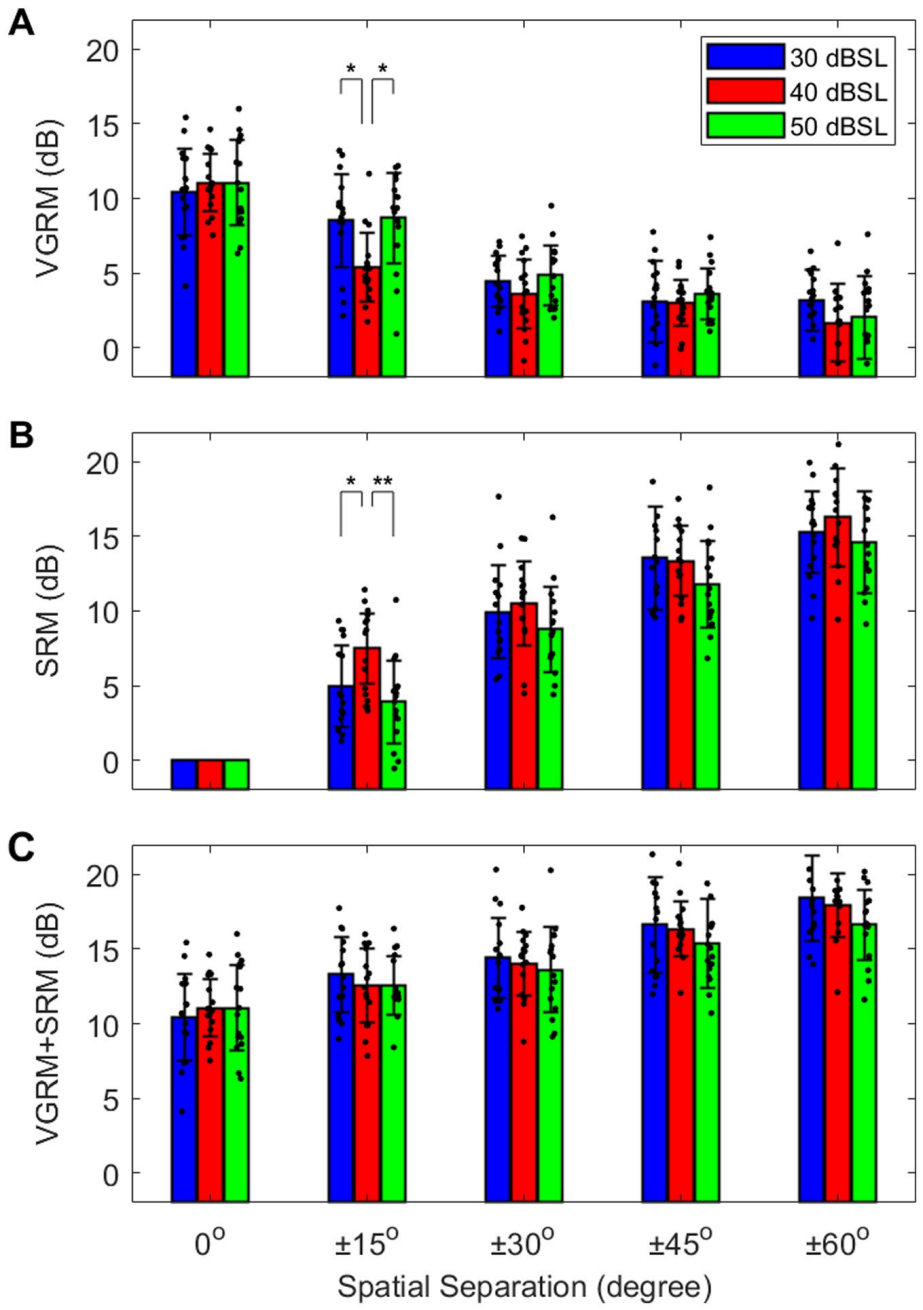
**(A)** Individual and average masking releases due to voice-gender difference (VGRM) and **(B)** spatial separation (SRM) between target and maskers, and **(C)** combined VGRM and SRM (VGRM+SRM) at different levels of the target levels (30, 40, and 50 dB SL). Error bars represent standard deviation of the mean. Asterisk symbols indicate significant differences (^*^p < 0.05; ^**^p < 0.01) between target level conditions.

Figure 3 shows three different types of masking release (i.e., VGRM, SRM, and VGRM + SRM) as a function of spatial separation for each target level. The average results show that all target level conditions elicited similar patterns of masking release changes. The VGRMs were maximized at 11 dB when the target and maskers were co-located (0°) and decreased by up to 10 dB as spatial separation increased to ±60°. Conversely, the SRM increased by up to 16 dB as spatial separation increased to ±60°. The masking release based on combined voice-gender difference and spatial separation (VGRM + SRM) increased by up to 8 dB from the 0° spatial separation to the ±60° spatial separation.

Masking release data were analyzed in each masking release type using LMM analyses with the amount of masking release (VGRM, SRM, or VGRM+SRM) as a dependent variable, the target-presentation levels (30, 40, and 50 dB), and the spatial separation (0°, ±15°, ±30°, ±45°, or ± 60°) as fixed effects, and the subject as a random effect. First, the results for VGRM showed significant main effects of both fixed factors (target level: *F*_2,210_ = 4.94, *p* = 0.009; spatial separation: *F*_4,210_ = 98.18, *p* < 0.001) and marginally significant interaction between two factors (*F*_8,210_ = 2.17, *p* = 0.051) was observed. *Post hoc* pairwise comparisons using Bonferroni correction were computed to better understand the interaction between those two fixed factors, especially for the target level. The results demonstrated that the VGRM at the 40 dB was significantly lower than the VGRMs at 30 and 50 dB only at the ±15° spatial condition (*p* < 0.05 for both cases). Second, the results of SRM also showed significant main effects of the two fixed factors (target level: *F*_2,210_ = 11.82, *p* < 0.001; spatial separation: *F*_4,210_ = 357.33, *p* < 0.001) and marginally significant interactions between those two factors (*F*_8,210_ = 3.37; *p* = 0.048). The pairwise comparison results demonstrated that the SRM at 40 dB was significantly higher than the SRMs at 30 and 50 dB only at the ±15° spatial condition (*p* < 0.05 for both cases). Third, the results for VGRM+SRM showed a significant main effect of spatial separation (*F*_4,210_ = 73.61, *p* < 0.001). That is, the target level did not change the overall VGRM + SRM performance.

## Discussion

The purpose of the current study was to investigate how the target presentation levels may influence the speech-on-speech masking outcome, especially the listener’s masking release performance, by two major acoustic cues (VGRM by talkers’ voice-gender differences and SRM by talkers’ spatial separations). The averaged results show that the VGRM was maximized at 11 dB when the target and maskers were co-located and decreased up to 10 dB as spatial separation increased to ±60°. Conversely, the SRM was maximized at 16 dB in the ±60° spatial condition. Those trends were consistent in all three target-level conditions. However, there were significant changes in the amount of VGRM and SRM at the 40-dB target condition, compared to the 30- and 50-dB targets, only at the ±15° separation between the target and maskers.

**Figure 4:**
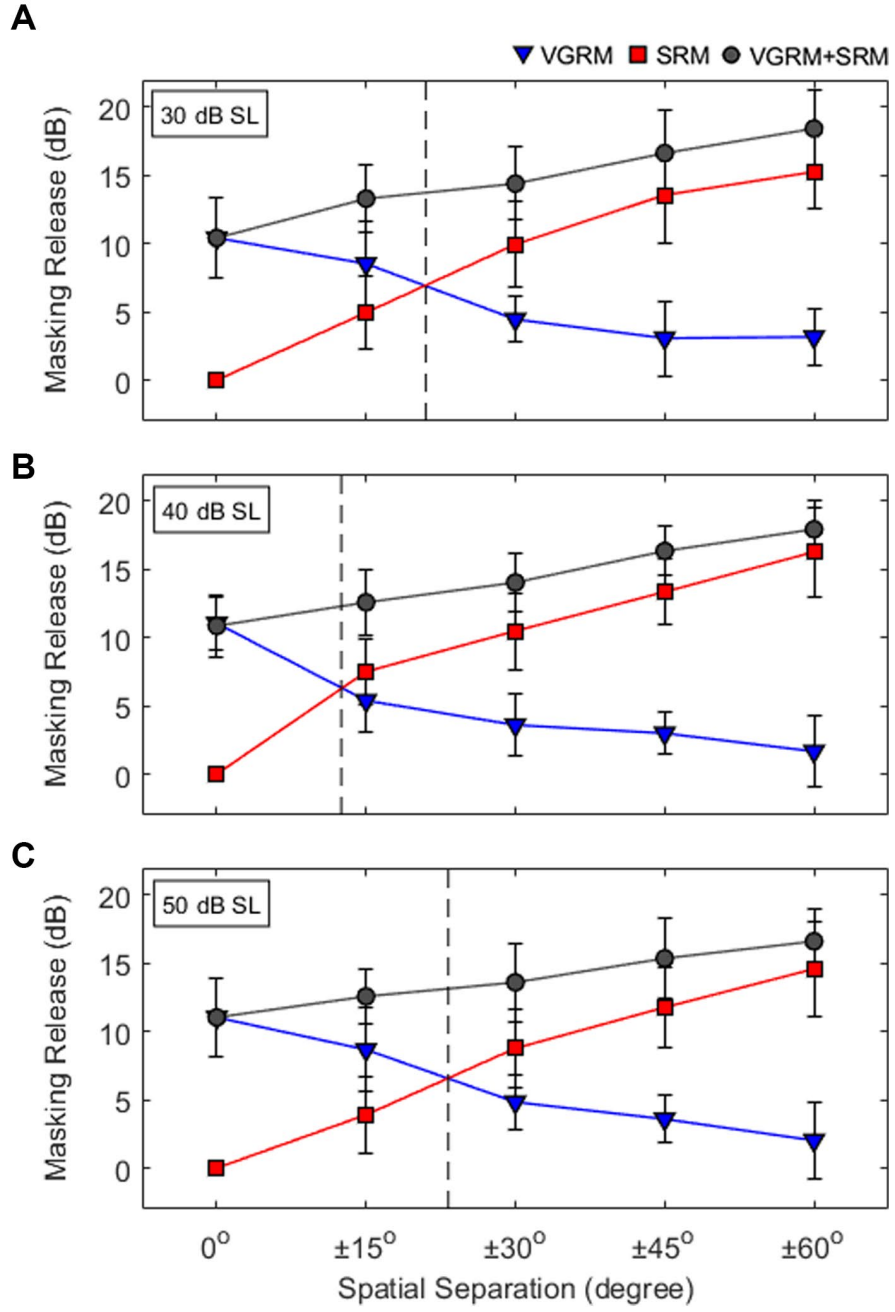
**(A-C)** Interactions of voice-gender release from masking (VGRM) and spatial release from masking (SRM) at different levels of the target levels (30, 40, and 50 dB SL). Vertical dashed lines indicate equal perceptual weighting between VGRM and SRM. Error bars represent standard deviation of the mean.

To understand those level-dependent changes in masking release, the VGRM and SRM data shown in Figure 3 have been plotted together at each target level condition (see Figure 4). The results show that when VGRM and SRM are mapped across a spatial field, the targets at the most comfortable presentation level (i.e., 40 dB SL) yield equal perceptual weighting between VGRM and SRM at a smaller spatial separation than those in quieter (30 dB SL) or louder (50 dB SL) target conditions. Here, equal perceptual weighting indicates a single point of intersection where the magnitude of masking release is the same for the two cue types, meaning that listeners rely on both cues equally in multi-talker listening situations. The results suggest that the weighting of masking release from two cues (i.e., VGRM and SRM) is non-linearly related to the target levels. Interestingly, those level-dependent changes of the masking release were only observed near the equal perceptual weighting points near ±15° spatial separations. In other spatial separations, the target levels did not change the masking release performance by either voice-gender difference or spatial separation cues (VGRM, SRM, and VGRM + SRM). This implies that a target presentation level can be one major factor associated with masking release performance. This is especially true when both voice-gender difference and spatial separation cues mostly have an equal effect on the listener’s speech-on-speech masking performance. In other words, the relative reliance on voice gender difference and spatial separation cues can be influenced by the target presentation levels when two cues are interchangeable.

The data from this study are consistent with the overall trends in masking release reported by previous studies using the CRM speech corpus (Ericson et al., 2004; Allen et al., 2008; Oh et al., 2021). Their data also revealed that those interchanges of perceptual weighting occurred when the target and maskers were presented at ±45° spatial separation and ~102-Hz F0 difference (Ericson et al., 2004), ±30° spatial separation and ~79-Hz F0 difference (Allen et al., 2008), and smaller than ±30° spatial separation and ~104-Hz F0 difference (Oh et al., 2021). It should be noted that the current study and Oh et al. (2021) found that the masking release was improved by up to 18 dB when talkers’ voice differences and spatial cues were incorporated, and the overall amount of masking release reported here was somewhat higher than that in other studies (~11 dB for Allen et al., 2008; ~6 dB for Ericson et al., 2004). Those discrepancies might be due to the differences in the study design for each study. First, both Allen et al. (2008) and Ericson et al. (2004) measured the amount of masking release at one spatial separation condition (±30° for Allen et al., 2008; ±45° for Ericson et al., 2004). Second, their studies used different stimulus presentation schemes. In the study by Allen et al. (2008), combined target and maskers were presented at a fixed 57 dB SPL with various TMR ranges. In the study by Ericson et al. (2004), the authors mentioned that all stimuli were presented at 70 dB SPL; however, there was no clear explanation about how the TMR levels were manipulated. In addition, the Ericson et al. study (2004) used a non-individualized Head-Related Transfer Function (HRTF) simulating talkers’ spatial separation. Third, both studies included small sample sizes (Allen et al., 2008: *N* = 5; Ericson et al., 2004: *N* = 7). Since the current study and the study of Oh et al. (2021) manipulated more target-masker spatial separations (0° to ±60°) with a larger sample size (the current study: *N* = 16; Oh et al., 2021: *N* = 20), the results from both studies may have provided more precise information on listener’s masking release performance by voice and spatial cues.

The results of this study raise some questions about why the target presentation levels had an impact on the VGRM and SRM in a non-linear manner only near their equal perceptual weighting points. Given the current data, it is difficult to explain a clear underlying mechanism. However, regarding the SRM, one potential mechanism may involve an interaction between the sensitivity to the binaural cues (interaural time and level differences) and the stimulus presentation level (e.g., Dietz et al., 2013). The findings in the current study of improved SRMs at small spatial separations (e.g., ±15°) at the most comfortable level might be due to a possible interaction between binaural sensitivity and target presentation level. Similar to the SRM, an interaction between the sensitivity to the monaural spectral cue (head-related transfer function) and the level may be involved in the VGRM. Another possible clue could be found in the study of Brungart (2001), which showed that varying target levels (48 to 85 dB SPL) at fixed masker levels (approximately 60–70 dB SPL) could yield a non-monotonic (“N”-shaped) psychometric function for the listener’s speech recognition performance when the target-masker gender combination was manipulated (e.g., informational masking). This implies that target sounds with lower or higher volumes relative to masker sounds may result in higher recognition thresholds than targets with medium volumes, or vice versa. As mentioned in the study of Brungart (2001), this could be due to the listener’s selective attention to the target voice which is softer or louder than the masker voices. Such a listener’s attentional factor might be able to explain non-linear changes in masking release performance due to talkers’ voice-gender differences (i.e., VGRM). In order to verify if the mechanisms proposed above apply, future studies will need to measure both binaural and monaural spectral cues by utilizing CRM stimuli at various target levels. Additionally, acoustical analyses of the stimuli with both the talker’s voice cue and spatial cue would be helpful. This approach could reveal an interaction mechanism between voice and spatial cues in speech-on-speech masking tasks since voice-gender difference and spatial separation could be interdependent features, allowing listeners to pay attention to a combination of cues rather than just one feature.

In summary, the findings in this study show that target presentation levels can vary speech-on-speech masking ability in NH listeners. Further research should be conducted to examine the effects of target presentation levels on speech-on-speech masking tasks for hearing-impaired (HI) listeners. Current hearing assistive devices utilize many speech enhancement features, such as noise-reduction strategies and directional microphones. Their features attempt to amplify the target signal, although some do not have the programming to differentiate it from noise and inturn increase the noise as well. For example, current hearing aid processing can differentiate and focus/amplify the target signal with directional microphones while simultaneously identifying and decreasing background noise. These processing strategies improve hearing-impaired listeners’ success with increased sound perception in quiet, but cannot overcome all difficulties in multi-talker situations such as the “cocktail party” phenomenon. Furthermore, HI listeners have smaller dynamic ranges than NH listeners, thus a small variation in target level may result in large changes in the masking release benefits by acoustic cues such as voice and spatial cues. Understanding these factors is essential for the development of effective program strategies for HI listeners, and this allows for more inclusivity for future use to promote a HI listener’s speech segregation performance in cocktail party environments, as our parameters include target levels allowing for those with hearing impairments to participate.

## Data availability statement

The raw data supporting the conclusions of this article will be made available by the authors, without undue reservation.

## Ethics statement

The studies involving humans were approved by the Institutional Review Board of University of Florida. The studies were conducted in accordance with the local legislation and institutional requirements. The participants provided their written informed consent to participate in this study.

## Author contributions

YO: Conceptualization, Data curation, Formal analysis, Funding acquisition, Investigation, Methodology, Project administration, Resources, Software, Supervision, Validation, Visualization, Writing – original draft, Writing – review & editing. PF: Writing – review & editing. JK: Writing – review & editing. GT: Data curation, Writing – review & editing. SB: Data curation, Writing – review & editing.
